# Evidence for ‘critical slowing down’ in seagrass: a stress gradient experiment at the southern limit of its range

**DOI:** 10.1038/s41598-018-34977-5

**Published:** 2018-11-22

**Authors:** El-Hacen M. El-Hacen, Tjeerd J. Bouma, Gregory S. Fivash, Amadou Abderahmane Sall, Theunis Piersma, Han Olff, Laura L. Govers

**Affiliations:** 1 Conservation Ecology Group, Groningen Institute for Evolutionary Life Sciences (GELIFES), University of Groningen, P.O. Box 11103, 9700 CC Groningen, The Netherlands; 2 Parc National du Banc d’Arguin (PNBA), Rue Gleiguime Ould Habiboullah, B Nord No. 100, B.P. 5355, Nouakchott, Mauritania; 3 NIOZ Royal Netherlands Institute for Sea Research, Department of Estuarine and Delta Systems and Utrecht University, P.O. Box 140, 4400 AC Yerseke, The Netherlands; 4 Institut Mauritanien de Recherches Océanographiques et des Pêches (IMROP), BP 22, Nouadhibou, Mauritania; 5 NIOZ Royal Netherlands Institute for Sea Research, Department of Coastal Systems and Utrecht University, P.O. Box 59, 1790 AB Den Burg, Texel The Netherlands; 6 Department of Aquatic Ecology and Environmental Biology, Institute for Water and Wetland Research (IWWR), Radboud University, Heyendaalseweg 135, 6525 AJ Nijmegen, The Netherlands

## Abstract

The theory of critical slowing down, i.e. the increasing recovery times of complex systems close to tipping points, has been proposed as an early warning signal for collapse. Empirical evidence for the reality of such warning signals is still rare in ecology. We studied this on *Zostera noltii* intertidal seagrass meadows at their southern range limit, the Banc d’Arguin, Mauritania. We analyse the environmental covariates of recovery rates using structural equation modelling (SEM), based on an experiment in which we assessed whether recovery after disturbances (i.e. seagrass & infauna removal) depends on stress intensity (increasing with elevation) and disturbance patch size (1 m^2^ *vs*. 9 m^2^). The SEM analyses revealed that higher biofilm density and sediment accretion best explained seagrass recovery rates. Experimental disturbances were followed by slow rates of recovery, regrowth occurring mainly in the coolest months of the year. Macrofauna recolonisation lagged behind seagrass recovery. Overall, the recovery rate was six times slower in the high intertidal zone than in the low zone. The large disturbances in the low zone recovered faster than the small ones in the high zone. This provides empirical evidence for critical slowing down with increasing desiccation stress in an intertidal seagrass system.

## Introduction

Seagrasses are effective ecosystem engineers^1^, creating habitats that support a broad biodiversity^2,3^. With ecosystem engineering involving a variety of positive feedbacks^4^, seagrass die-off events often follow alternative stable state dynamics that by their nature can be difficult to reverse^5^. Unfortunately, over the last decades, several sudden landscape-scale seagrass die-offs have been reported. This concerns the Wadden Sea of The Netherlands^6^, Spencer Gulf, Australia^7^, different part of the Mediterranean Sea^8,9^, Odense Fjord, Denmark^10^, Florida Bay, USA^11^, Chesapeake Bay, USA^12^, Jangheung Bay, Korea^13^, and Banc d’Arguin, Mauritania^14^. These die-off events have been attributed to hypersaline conditions^15,16^, extreme temperature^7,17^, and sulphide toxicity^14,18^. The future of seagrass beds is dependent, to a large extent, on our ability to understand and predict seagrass recovery following large-scale die-off events within the framework of climate change related stresses such as sea level rise and extreme weather conditions.

The speed at which seagrass meadows may recolonise gaps caused by die-offs is a crucial component of their long-term persistence^19^, and determines the frequency at which perturbations may occur without resulting in a regime shift toward an alternative ecosystem state^5,20^. Different indicators have been suggested to predict critical thresholds before regime shifts, including ‘critical slowing down’ in responses to adverse environmental conditions^21–23^. Critical slowing down implies that when an ecosystem approaches a tipping point, it will show increasingly slower recovery rates following a disturbance^22,24,25^. Experimental evidence for the occurrence of critical slowing down, however, is still rare in ecology especially for natural, intact ecosystems (but see^23^).

Here, we studied the potential for critical slowing down to act as an indicator for collapse in seagrass *Zostera noltii* at the southern limit of its range: the subtropical intertidal flats at the Banc d’Arguin, off the Mauritanian coast^26^. At Banc d’Arguin, *Z. noltii* covers most of the 500 km^2^ of intertidal flats bordering the Sahara, encountering more extreme environmental conditions than at temperate zones^27^. The seagrass may experience large temperature fluctuations (i.e., 11–37 °C; unpub. data), hypersaline conditions (i.e., 38–54.5‰^28^), intense dust storms (up to 100 events/year^29^), and rather frequent heat-waves (40–60 days/year with air temperature exceeding 41 °C^30^). Living in such extreme conditions may make seagrass here vulnerable to further exacerbation of climate conditions^31^. Despite their rather pristine state, natural mass-mortalities have been observed over the last couple of years (^14^; Supplementary 1; Fig. S1). It has been suggested that these die-offs are the result of a breakdown of feedback relationships between *Z. noltii* and its most important mutualistic partner, the sulphide-consuming^32^ and nitrogen-fixing^33^ lucinid bivalve *Loripes orbiculatus*. In this system, landscape-level die-offs occur especially high on the intertidal elevational gradient, while lower, longer inundated seagrass beds are much less sensitive to this^14^.

Seagrass recovery after disturbance is affected by various biota and abiotic conditions. High porewater sulphide concentrations are toxic to seagrass^18,34^ and may negatively affect recovery following die-off. Sediment dynamics have been shown to affect *Z. noltii* recovery in an experimental study assessing the effect of intertidal ecosystem engineers on seagrass responses to disturbance^35^. Finally, other sediment characteristics such as water content and grain size have been identified to play an important role in *Z. noltii* dynamics^36^. To study how fast the seagrass beds recover from different-sized disturbances, we therefore set up an experiment on an intertidal flat in Banc d’Arguin at different elevations. Specifically, we aimed to assess (1) which ambient abiotic and biotic factors might influence variability in recovery rates, (2) whether *Z. noltii* exhibits critical slowing down following a sudden die-off event at different stress levels (i.e. inundation height), and (3) if critical slowing down is a function of the disturbance-scale. We expected high sulphide concentrations and high rates of sedimentation to slow down the recovery of seagrass in the disturbed plots, while sediment moisture and organic matter contents, as well as the abundance of the lucinid bivalve *Loripes orbiculatus*, should speed up recovery.

## Methods

### Study site

**Figure 1:**
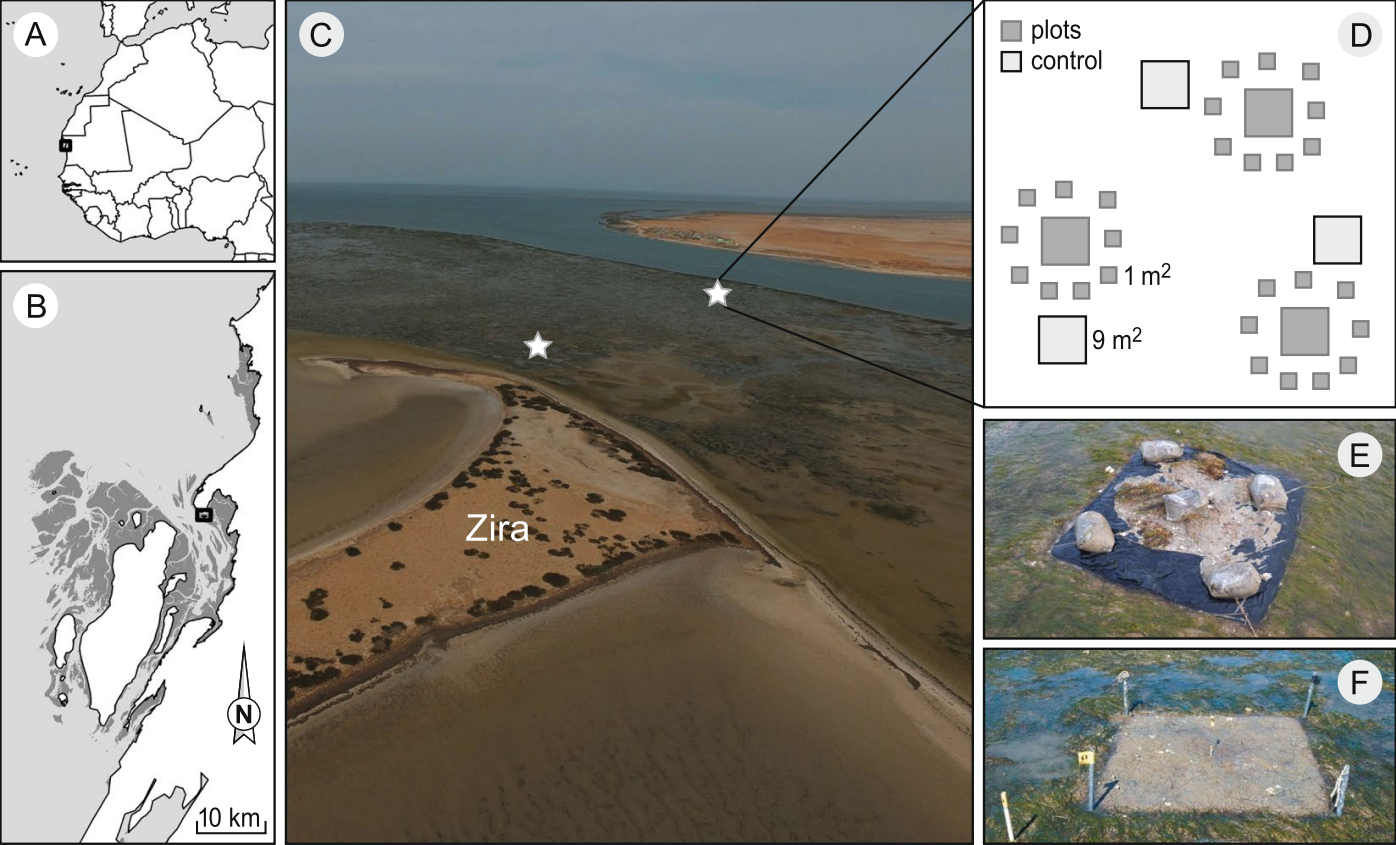
(**A**,**B**) Maps of the study area and (**C**) aerial photo showing the two experimental sites chosen on an elevational gradient next to the islet of Zira within the Parc National du Banc d’Arguin, Mauritania. (**D**) A schematic representation of the experimental design: three replicate blocks were established at each site, and consisted, each, of one large (9 m^2^) and nine small die-off (1 m^2^) treatments as well as one large (9 m^2^) control. (**E**) Photo demonstrating the technique used to induce seagrass mortality within plots. (**F**) The status of the die-off plot at the start of the monitoring program. Dark grey in the maps represents intertidal flats, light grey shows the ocean, and the white depicts the land. Maps were created in Esri ArcMap 10.4 (http://desktop.arcgis.com/en/arcmap/) based on Landsat imagery (NASA, scene of February 1, 2016) provided at no costs by USGS^89^ at: http://earthexplorer.usgs.gov/. Aerial photograph courtesy Laura Soissons.

The study was carried out on the mudflats surrounding the islet of Zira (19°52′17.05″N, 16°17′49.51″W; Fig. 1A,B) in the Parc National du Banc d’Arguin (PNBA), Mauritania. PNBA is the largest marine protected area in Africa, and covers 12000 km^2^ (half marine and half terrestrial). The marine part is characterised by a complex, but shallow, bathymetry and comprises 500 km^2^ of intertidal flats covered with seagrass, especially the intertidal *Zostera noltii*, but also *Halodule wrightii* and subtidal *Cymodocea nodosa* for more than 80%^28^. PNBA, so far, is still to a large extent a pristine environment^37^.

The climate of the study region consists of a distinct warm season (June-September)^30^. Wind is predominantly a northern trade-wind and there is hardly any precipitation in the area year-round^38^. Salinity is generally high due to the isolated nature of the inner intertidal part of PNBA, and could reach extreme values (>80‰) in the locked bays^39^.

### Experimental set-up and sampling procedures

To assess the recovery potential of seagrass, a die-off experiment was performed at two sites along an intertidal elevation gradient within the same continuous meadow (Fig. 1C). At both sites, experiments were set-up in three replicate blocks of small (1 * 1 m, 9 per block) and large (3 * 3 m, 1 per block) disturbed plots, and large (3*3 m, 1 per block) controls (Fig. 1D). This design kept the total disturbed areas for small and large plots the same. Small plots were placed in a circular radius surrounding the large plot in each repetition, and controls were located just outside the radius of small plots (Fig. 1D). Plots were placed at least 6 m apart to reduce unwanted artefact effects of the clearings on the plots. The die-offs were enforced by placing two layers of plastic tarps over the plots for two weeks from 19 January to 5 February 2015 (Fig. 1E). This led to 100% mortality of the seagrass (Fig. 1F). After the removal of the tarps, the total surface area covered by seagrass tissue (% seagrass cover) was visually estimated at plot level (1 m^2^ for small plots and 9 m^2^ for the larger ones) on an approximately monthly basis. Small plots cover estimations were done with the aid of a 1*1 m frame divided into 10*10 cm quadrats. Total seagrass area (mm^−2^) per plot was then computed from the percentage cover estimates. Monthly recovery rate (“clearing contraction rate”: RR, mm day^−1^) was calculated following^35^ as:

$$RR=\frac{\sqrt{({X}_{t1})-\surd {X}_{t2}}}{2{\rm{\Delta }}t}$$

where *X*_*t1*_ and *X*_*t2*_ are plot bare area at the start and end of the measurement period, respectively, and t is the number of days between t1 and t2.

The recovery of the biomass of seagrass and the benthic community (i.e. to characterise parameters that may affect seagrass recovery), was assessed by a rotation sampling protocol. This helped us to avoid re-sampling the same location and minimised disturbance that might affect the recovery process. A benthic core of 7 cm diameter was taken in half of the small disturbed plots (n = 4) plus all the large disturbed and control plots (n = 1) in each experimental block every six months, the other half of the small plots was sampled six months later. Each plot was divided into 4 sub-plots (50*50 cm for the small plots and 1.5*1.5 m for the large ones), and each sub-plot was sampled once during the study period. The resulting pits from the coring were filled with sediment from similar nearby habitat. Benthic fauna (sieved through 1 mm mesh) was sorted and all the bivalves and gastropods specimens were identified to the species level and their length measured to the nearest 0.1 mm. Polychaeta and Crustacea were identified to the family level. Benthic ash-free dry biomass (AFDM, loss of ignition at 560 °C for three hours) was determined per plot (with a precision of ±0.0001 g) after oven drying at 60 °C for two days to reach a constant weight. Seagrass above- and below-ground biomass was dried until constant weight at 70 °C for 48 h, and weighed with a precision of ±0.01 g.

To further characterise parameters that may affect seagrass recovery, the following environmental variables were measured at a six month interval over two years: porewater sulphide concentrations^18^ inside the plots were sampled with vacuumed syringes connected to ceramic soil moisture samples (Eijkelkamp Agrisearch Equipment, Giesbeek, the Netherlands) at 5 cm depth in the sediment, and stored in vacuum-sealed syringes (see^40^). Within 4 hours after sampling, sulphide levels were then measured in the laboratory in a solution of 50% porewater sample, 50% sulphide anti-oxidation buffer using a calibrated Hanna (Italy), HI 4115 silver electrode. Redox potential (mVolt)^41^ was measured at 5 cm depth using five Pt electrodes and one HgCl/KCl reference electrode connected to a GL220 Data logger (Graphtec GB Ltd., Wexham, UK). The mean of its five Pt electrode readings were calibrated using a known standard hydrogen electrode. Biofilm (diatoms, cyanobacteria, and green algae) densities (µg.cm^−2^)^42^ were measured using the instrument BenthoTorch (bbe-Moldaenke BenthoTorch, Germany).

Sediment dynamics (erosion, accretion)^43^ was assessed at two stages: (1) Plot surface elevations (bed level) were measured in May 2015 (six months after the start of the experiment) with the real time kinematic global positioning system (RTK-GPS; Trimble, California, United States). (2) Net sediment accretion was estimated between January 2015 and January 2016 with ‘Erosion’ pins^44^. Other sediment characteristics were measured once including sediment moisture content (%)^36^ using 35.34 cm^−3^ volumetric samples dried at 105 °C for 72 h, and organic matter content^36^ (OM, loss of ignition at 500 °C for four hours).

### Statistical analyses

All statistical analyses were performed with the free statistical software R version 3.4.3^45^. Data exploration following^46^ indicated severe zero inflation in the sulphide data of Jan-2016, May-2016, and Jan-2017, and hence sulphide data collected during these dates were not considered in the analyses.

The biophysical setting that may have affected the seagrass recovery was assessed as follows. Initial linear mixed-effects modelling with blocks as random-effects revealed no significant effect of Blocks but a significant three-way interaction between sampling date, die-off treatments and elevation. Block effects were therefore not considered in subsequent analyses. As 3-way interactions are difficult to interpret, the data were further analysed with 2-way ANOVAs for each sampling period separately. For this, two-way analysis of variance (ANOVA) was applied to determine whether there were significant differences (*P* < 0.05) between die-off treatments along the elevational gradient (low *vs*. high) on (*i*) porewater sulphide concentrations; (*ii*) sediment moisture content; (*iii*) net sediment accretion; (*iv*) sediment redox potential; and (*v*) biofilm densities. Tukey’s honest significance difference (HSD) post-hoc test was used for multiple comparisons of means at a 95% confidence interval. Normality and heteroscedasticity of data were inspected visually on the residuals. Sulphide concentrations were square-root transformed to meet parametric assumption.

Second, structural equation modelling (SEM^47,48^); was performed to describe the most likely structure of the set of predictor variables affecting the seagrass recovery using the entire dataset, including all die-off treatments in both elevational zones. SEMs were constructed using piecewiseSEM package in R (https://github.com/jslefche/piecewiseSEM/tree/2.0)^49^, which allows the fitting of mixed-effect models and a hierarchical design. We selected this method because recovery rates, as well as the measured abiotic and biotic variables included in the SEM, were temporally and spatially autocorrelated, and thus required mixed-effects modelling. Models were fitted with blocks and sampling dates as random effects, and an additional autoregressive moving average (ARMA) correlation structure with a six-months lag to account for repeated measures autocorrelation^50^. To study the impact of environmental conditions (measured at six month intervals) on seagrass recovery trajectory (measured at monthly intervals), recovery rate was averaged for the five months preceding each of the environmental measurements.

The SEM analysis was conducted in three stages. First an overall *a priori* model of interactions based on knowledge from previous studies on seagrass functioning in the area^14,27,32,51^ was created, a model which included all relevant biotic and abiotic factors (Fig. S2). Next, the resulting interactions were translated into lists of structured equations, and finally these equations were evaluated against the observed data to support or reject the hypothesised causal structure of the predictor variables. Sediment bulk density and water content were not included in the analysis due to their high collinearity with sediment organic matter. Correlations among the remaining variables (<0.61) were considered acceptable^52^. Model fits were determined using Fisher’s C statistic and coefficients of determination (R^2^) values^49^. To meet the homogeneity of variance and linearity assumptions, all variables were log transformed except benthos AFDM, which was square-root transformed. Control plots had a mean and variance recovery rate of zero (i.e., no change) and were excluded from the SEM analysis.

Linear mixed-effects modelling (LMER) using restricted maximum likelihood fitting was done with the lme4 package in R^53^, in order to investigate the effect of die-off treatments (control, small, large) and elevational gradient (high, low) on the monthly percentage cover estimates in the disturbed plots. Die-off treatments and elevational gradient were included as fixed-effects and date and block as random-effects. Model selection was carried out with backward selection procedure based on reduction of Akaike’s information criterion (AIC). *P*-values from F tests were calculated with the lmerTest package^54^ using Satterthwaite’s approximation of the denominator degrees of freedom. Pairwise comparisons were obtained using the Tukey test in the LSMEANS package^55^ and the final model was validated by inspecting the residuals. Percentage cover data were arcsine square-root transformed to improve homogeneity of residual variance.

Macrofaunal recovery was assessed on samples taken 6, 12, 18, and 24 months after defaunation by comparing assemblages in the disturbed plots to those of the controls. Differences in the composition in macrobenthic assemblages in the treatments were first assessed using non-metric multidimensional scaling (nMDS) based on Bray-Curtis similarity. Then, a one-way analysis of similarity (ANOSIM) was performed to test the significant differences in macrobenthic assemblages between the die-off treatments grouped within the elevational zones to create one response variable. Complete benthic recovery was considered when no-significant difference in assemblages was detected between defaunated and control plots. All multivariate analyses were performed using vegan package in R.

## Results

### Changing biophysical contexts during seagrass die-offs

**Figure 2:**
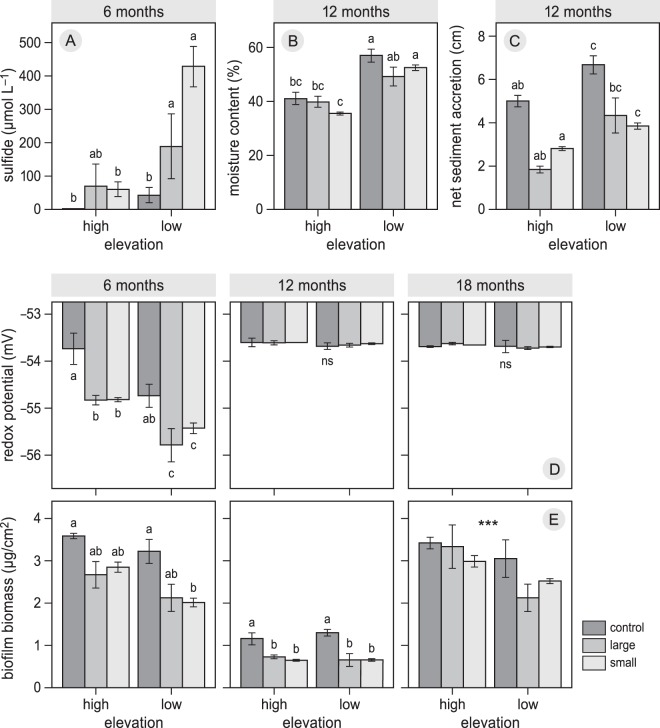
The effects of die-off treatments (control, large, small) along an elevational gradient (high, low) on (**A**) porewater sulphide concentration (µ mol L^−1^), (**B**) sediment moisture content (%), (**C**) net sediment accretion (cm), (**D**) sediment redox potential (m V), and biofilm density (µg cm^−2^). Bars represent means ± SE; different lowercase letters indicate a significant deference computed for each month separately (Tukey HSD, *P* < 0.05). (***) Sign in May 16 of the panel (**E**) represents the significance (*P* < 0.001) between zones.

Over the six months following the die-off treatments, plot size and elevation did affect significantly, non-interactive, porewater sulphide concentrations (Table 1, Fig. 2A), with significantly higher sulphide concentrations in the disturbed plots than the controls and in the low zone than in the high zone (Table 1, Fig. 2A). Similarly, plot size and elevation also significantly modified other sediment characteristics. Sediment moisture content, sediment accretion, and sediment redox potential were lower in disturbed plots than in controls, and marginally lower for water content (Table 1, Fig. 2B–D). Water content and sediment accretion were both significantly higher in the low zone than in the high zone, while redox potential was lowest in the low zone (Table 1, Fig. 2B–D). During the second year after die-off (12 and 18 months) redox potential did not differ either between plot sizes or zones (Table 1, Fig. 2D).

**Table 1:** Results of the analysis of variance (two-way ANOVA) of the mean effects of die-off treatment (control, large, and small) along an elevational gradient (high, low) on the porewater sulphide concentration, sediment moisture content, net sediment accretion, sediment redox potential, and biofilm densities. Bold characters indicate significant effects.

Source of variations	df	*MS*	*F*	*P*
**Sulphide**				
June-2015				
Treatments	2	251	8.3	**<0.01**
Zone	1	1386	45.6	**<0.001**
Residuals	36	30.4		
**Moisture**				
June-2015				
Treatments	2	68	2.7	0.065
Zone	1	4326	171.8	**<0.001**
Residuals	60	1510		
**Sedimentation**				
May-2016				
Treatments	2	17.66	32.5	**<0.001**
Zone	1	25.47	46.9	**<0.001**
Treatments*Zone	2	1.55	2.8	0.06
Residuals	60	0.54		
**Redox potential**				
June-2015				
Treatments	2	2.3	10	**<0.001**
Zone	1	7.5	32.5	**<0.001**
Residuals	60	0.23		
Jan-2016				
Treatments	2	0.003	0.5	0.6
Zone	1	0.01	2.31	0.1
Residuals	60	0.0006		
May-2016				
Treatments	2	0.002	0.02	0.9
Zone	1	0.02	2.6	0.1
Residuals	60	0.0007		
**Biofilm density**				
June-2015				
Treatments	2	2.6	7.3	**<0.01**
Zone	1	9.7	27.5	**<0.001**
Residuals	60	0.3		
Jan-2016				
Treatments	2	0.9	33	**<0.001**
Zone	1	0.0008	0.02	0.8
Residuals	60	0.02		
May-2016				
Treatments	2	0.64	1.83	0.1
Zone	1	4.5	12.8	**<0.001**
Residuals	60	0.3

Overall, biofilm densities were lower in the disturbed plots than the controls in both elevational zones, with a general decrease in winter compared to spring (Table 1 and Fig. 2E). Mean biofilm densities did not significantly differ between zones in winter, but in spring densities were lowest in the low zone (Table 1; Fig. 2E). Sediment organic matter contents did not differ significantly between die-off treatments (Table 1) but differed significantly between the low (mean = 6.6, se 0.24) and the high (mean = 2.8, se 0.08) zones (Table 1). Finally, bed levels in the high zone significantly decreased (Table 1) for both the large (mean = 1.17 cm, se 0.16) and small (mean = 0.83, se 0.07) disturbances compared to the controls of the same block 6 months after the start of the experiment. Similarly, bed levels in the low zone decreased significantly for the large (mean = 0.88, se 0.16) and the small (mean = 0.65, se 0.1) disturbances compared to the controls of the same block. The main effect of elevational zone on bed level was not significant (Table 1).

### Recovery trajectory: what are the key biophysical covariates?

**Figure 3:**
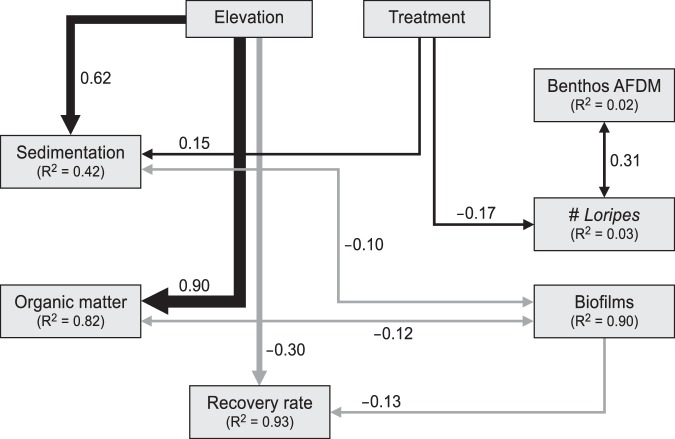
Final piecewise structural equation model (Fisher’s C statistic = 14.65, *P* = 0.56), representing the effects of elevation, die-off size, and various abiotic and biotic variables on the recovery rate of *Zostera noltii* after disturbance. Solid lines indicate significant paths (*P* < 0.05); nonsignificant relationships were omitted for clarity. Hypothesised causal relationships (one-headed arrows) were weighed with standardised path coefficients, while the double-headed arrows were weighed by the covariance between connected variables. The thicknesses of the significant paths are proportional to the magnitude of the standardised regression coefficient. Numbers between brackets represent coefficients of determination (R^2^) related to the variable. Black arrows represent positive paths, and grey ones are indicative of negative relationships.

The piecewise SEM model fitted the observed data very well (Fisher’s C statistic = 14.65, *P* = 0.56), and revealed that only elevation and biofilm biomass directly affected recovery rate, but not die-off size (Fig. 3). However, both elevation and die-off treatments were indirectly associated with recovery rate through their effect on sedimentation and OM, which both had a strong effect on biofilms (two directional relationship; Fig. 3). As expected, recovery rate was negatively correlated to elevation (Fig. 3). Of all measured environmental variables, biofilm densities had the strongest negative effect on seagrass recovery (Fig. 3). Sedimentation and OM were significantly related to elevation, while die-off treatments had only a significant effect on sedimentation and the number of *Loripes orbiculatus* (Fig. 3).

Macrobenthic recolonisation in the defaunated plots were manifested by a gradual increase in total abundance and biomass (Fig. S3). MDS ordination showed clear variation in benthic assemblages between disturbed plots and controls (Fig. S4). This pattern was confirmed by ANOSIM results, which revealed consistence significant differences between disturbed plots and controls over time (ANOSIM: 6 months, *R* = 0.24, *P* = 0.001; 12 months, *R* = 0.18, *P* = 0.019; 18 months, *R* = 0.14, *P* = 0.02; 24 months, *R* = 0.22, *P* = 0.007). Differences between controls and disturbed plots decreased over time although recovery was not complete over the 24 months of monitoring, even in the small plots in the low zone that had complete seagrass recovery by the time.

### Critical slowing down along a desiccation gradient

**Figure 4:**
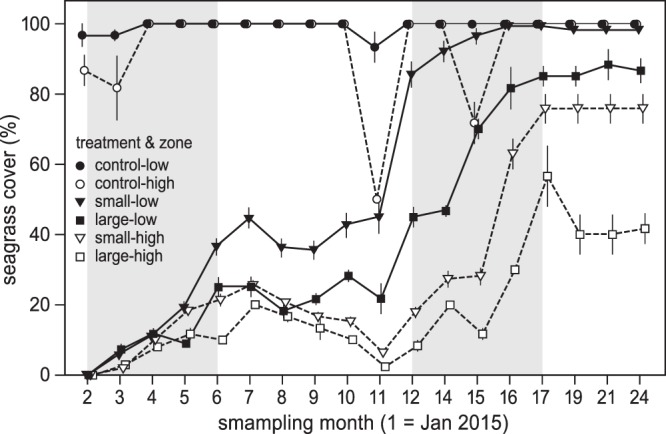
Relative change in *Zostera noltii* cover of the different die-off treatments (small, large, control) over 2015 and 2016 along an intertidal gradient (high and low) in Banc d’Arguin, Mauritania. Open symbols with dashed lines represent the high zone, while the filled symbols with solid lines represent the low zone. Values are means ± SE. Grey areas represent winter and spring months, white areas summer and fall months.

All experimental die-off plots showed gradual recovery towards the pre-disturbance cover but with different success. Recovery occurred from the edge of the plots toward the inside by clonal propagation. No recovery by means of sexual regeneration (seed) was observed. The different elevational zones were very different in final recovery. Recovery in the low zone was almost complete while none of the high zone plots recovered completely over 24 months of monitoring. Seagrass recovery varied significantly between the scales of disturbance (LMER: *F*_(2, 1161)_ = 558.5, *P* < 0.001, Fig. 4) and between elevational zones (LMER: *F*_(1, 6)_ = 39.77, *P* < 0.001, Fig. 4), with increasing recovery time with increasing elevation and disturbance size (Table 2; Fig. 4). A significant interaction (LMER: *F*_(2, 1161)_ = 17.88, *P* < 0.001) between elevation and disturbance size was evident: the recovery time of the small disturbances of the high zone was slower than the large disturbances of the low zone (Table 2; Fig. 4). Recovery seems to have taken place mainly in winter and spring, while no clearing contraction observed in summer and fall (Fig. 4). During the growing season (winter and spring), the recovery rate of the high zone was, on average (±se) 0.1 ± 0.02 mm day^−1^ in the small plots and 0.03 ± 0.1 mm day^−1^ in the large plots, while in the low zone it was 0.51 ± 0.1 mm day^−1^ for the small plots and 0.23 ± 0.01 mm day^−1^ for the large plots. On average recovery in the high zone was 6.38 times slower than in the low zone. The observed collapses in seagrass cover, especially in the high zone, over the 11 and 15 months (Fig. 4) coincided with mass-sediment deposition events in the area.

**Table 2:** Tukey’s all pairwise comparisons of seagrass recovery responses to die-off treatments (control, large and small) and elevational gradient (high, low) following linear mixed-effects models (LMER). Significant findings highlighted in bold.

Contrast	Estimate	SE	df	t-value	*P*-value
High, large *vs*. Low, large	−0.27	0.06	26	−4.93	**0.0005**
High, large *vs*. High, small	−0.16	0.03	1161	−4.79	**<0.0001**
High, large *vs*. Low, small	−0.53	0.05	13	−11.42	**<0.0001**
Low, large *vs*. High, small	0.11	0.05	13	2.39	0.2273
Low, large *vs*. Low, small	−0.26	0.03	1161	−7.59	**<0.0001**
High, small *vs*. Low, small	−0.37	0.03	4	−10.46	**0.0021**

## Discussion

At Banc d’Arguin, Mauritania, *Zostera noltii* grows at the southern limit of its distribution range, which enabled us to empirically study critical slowing down signals along a desiccation gradient in a system prone to desiccation stress^14^. Our experiment demonstrated slower recovery higher in the intertidal, i.e. at sites with increased desiccation stress. Combined with the results from previous work in this system^14^, this indicates that seagrass growing higher on the elevational gradient is closer to a tipping point. This critical slowing down may indicate the systems vulnerability to desiccation stress and extreme weather events due to global warming.

Previous work in this ecosystem suggests that bare intertidal flats dominated by microphytobenthos can constitute an alternative stable state to seagrass^56,57^. Indeed, Structural Equation Modelling showed that biofilm densities negatively affected seagrass recovery rate (Fig. 3). Due to their ecosystem engineering effects on sediment characteristics, cyanobacteria or diatom biofilms can exclude seagrasses and dominate benthic primary production^56^. In our study system, microphytobenthos layers potentially seal sediment-air interface through the excretion of extracellular polymeric substances (EPSs)^58^, and leading to unfavourable growing conditions (anoxic, high sulphide concentrations) for *Z. noltii*. We suggest that this represents an under-studied topic. While opportunistic macroalgae are known to outcompete seagrass beds in eutrophic systems^4,59,60^, less attention has been dedicated to the microphytobenthos (MPB) communities which often dominate soft-sediments and could represent a later stage of the succession from vegetated to bare^61^. Even though all the die-off plots showed gradual recovery, our findings nevertheless suggest that biofilm layers can have a significant negative effect on seagrass recovery and may be responsible for an alternative stable state characterised by bare sediment. Apparently, the 3*3 m die-off plots were not large enough to create permanent alternative microphytobenthos dominated states, suggesting once again that this is strongly scale-dependent^62–65^.

An unexpected outcome of our experiment is that the seagrass recovery was independent of benthic community composition and seems to have occurred in the near-absence of the sulphide-consuming lucinid bivalves, *Loripes orbiculatus*, despite the previously shown importance of this bivalve for *Z. noltii* under high sulphide conditions^32,33^. It could be concluded that seagrass, in our study site, could colonise new patches without the help of the lucinid bivalves. The long-term survival and resilience of these patches, however, may well be dependent on the symbiosis with *Loripes*^14,32^, especially during sulphide pulses that apparently did not occur during our study period.

The very slow recovery was remarkable for a fast-growing seagrass species with high rhizome expansion rate as *Z. noltii*^19,66^. Different studies showed that *Z. noltii* can fill in small clearings (<1 m^2^) within a month after disturbance^43^. Related species in the subtropics as *Zostera capricorni*, are also known to quickly recolonise clearings created by grazing dugong (*Dugong dugon* Müller) within a year after disturbance^67^, while *Halodule wrightii*, a species that coexist with *Z. noltii* at Banc d’Arguin, has been shown to recover within 9 months from small (0.25 m^2^) perturbations^68^. The remarkable slow recovery of *Z. noltii* at the edge of its distribution may indicate low overall resilience. Recovery occurred mainly in spring and winter and the species’ growth seems to slow down in the warmer summer and autumn. Although the growth in spring might overlap with the reported growth season for *Z. noltii* worldwide^69,70^, winter growth and expansion has, to our knowledge, not been reported before in this intertidal species. This suggests that with increasing temperature, the species could shift growing periods to cooler seasons. Adjusting phenology in response to adverse temperature (cold and warm) is well established in dendrology^71–73^ and has been suggested for *Zostera marina*^74,75^.

Although the observed recovery times are longer than the recovery times reported for other seagrasses occurring in the tropic^76^, the expansion strategy fit the most common one reported for the tropical and subtropical seagrasses, asexual recolonisation^76,77^. Throughout the recovery period, clearing contraction happened only from the edges toward the centre, which is indicative of asexual (rhizome elongation) rather than sexual (seed establishment) expansion. Small-scale die-offs facilitate asexual recovery with an increase in the edge to area ratio which favours neighbouring rhizomes to expand toward the bare patches. The expansion strategy of the species may have important consequences for the recovery from die-off events, especially for isolated intertidal flats without physical connections with other seagrass meadows.

The present study adds experimental support to the theoretical analyses showing a critical slowing down response when natural systems are approaching tipping points and regime shifts^21,22,78–80^. Empirical evidence for critical slowing down theory is still scarce in ecology, especially at the landscape scale (but see^23,80^). This slowing down recovery criterion might be of great importance for seagrass management and monitoring now that these habitats are under such pressure^5,81–83^. Critical slowing down has been mathematically proposed for seagrass before^84^, and been used as a potential indicator for impending seagrass meadow collapse^85^. At Banc d’Arguin, the slowing down along the elevational gradient is likely to manifest itself in an elevation-related loss of resilience and a decreasing capacity of the higher intertidal flats to withstand disturbances. At the highest zone, the larger clearing still had not recovered 3.5 year after the disturbance (pers. obs.), indicating that we may have been close to tipping the system to another state.

We observed that the recovery time was faster for the large disturbances in the low zone than the small disturbances of the high zone, and that a single large-scale disturbance will have a larger impact than a disturbance of similar extent but spread out over smaller areas. This has major implications on how to design critical slowing down tests. The great contrast in recovery rates between the different sized treatments along the gradient implies that, when studied in small-sized plots, critical slowing down will be underestimated while the resilience will be overestimated. Elsewhere, it has been shown in an experimental clearing that *Z. noltii* recovery is vigorously scale dependent, and was mediated by the ecosystem engineers around^35^. Critical slowing down assessments traditionally ignore disturbance sizes (but see^86^) and often use the notion of recovery from small disturbances^22,24^, which leaves an important gap in our understanding to this useful evaluation tool. Nonetheless Dai *et al*.^79^, have introduced the term ‘recovery length’ as a connectivity distant indication for population recovery. The perturbation size, especially edge to area ratio, is known to affect seagrass recovery time^87,88^ and its inclusion into critical slowing down assessments will further improve our understanding and prediction to the future of seagrasses.

The present study presents empirical evidence for a critical slowing down response in a model seagrass species (*Z. noltii*) along a desiccation gradient at the southern edge of its range. The results revealed that the *Z. noltii* in Banc d’Arguin has a low capacity to recover after die-off events, providing a clear sign that these meadows are on the verge of tipping points especially higher on the intertidal gradient. The die-off experimental outcomes illustrated that the recovery was size-dependent and identify perturbation size as a new dimension that should be considered for future critical slowing down assessments. Finally, assessing critical slowing down along intertidal elevation may provide a good indication of vulnerability of seagrass to desiccation stress and extreme weather events due to global warming.

## Electronic supplementary material

Supplementary information

## Data Availability

Data supporting the findings of this study are available from the authors and will be made archived and publicly available in the University of Groningen Research Data Repository (http://www.rug.nl/research/gelifes/research/data-management/repository?lang=en).
