# Supplementary material for: Evidence for ‘critical slowing down’ in seagrass: a stress gradient experiment at the southern limit of its range
Source: Sci Rep. 2018 Nov 22;8:17263. doi: 10.1038/s41598-018-34977-5 (PMC6250700; doi:10.1038/s41598-018-34977-5)
Supplement: Supplementary file 1 — Supplementary information [file 41598_2018_34977_MOESM1_ESM.docx]

# Supplementary information to: Evidence for ‘critical slowing down’ in seagrass: a stress gradient experiment at the southern limit of its range

El-Hacen M. El-Hacen^1, 2,*^, Tjeerd J. Bouma^1, 3^, Gregory S. Fivash^1, 3^, Amadou Abderahmane Sall^4^, Theunis Piersma^1, 5^, Han Olff^1^ and Laura L. Govers^1, 6^

^1^ Conservation Ecology Group, Groningen Institute for Evolutionary Life Sciences (GELIFES), University of Groningen, P.O. Box 11103, 9700 CC Groningen, The Netherlands

^2^ Parc National du Banc d’Arguin (PNBA), Rue Gleiguime Ould Habiboullah, B Nord No 100, B.P. 5355, Nouakchott, R.I. de Mauritanie

^3^ NIOZ Royal Netherlands Institute for Sea Research, Department of Estuarine and Delta Systems and Utrecht University, P.O. Box 140, 4400 AC Yerseke, The Netherlands

^4^ Institut Mauritanien de Recherches Océanographiques et des Pêches (IMROP), BP 22, Nouadhibou, R.I. de Mauritanie

^5^ NIOZ Royal Netherlands Institute for Sea Research, Department of Coastal Systems and Utrecht University, P.O. Box 59, 1790 AB Den Burg, Texel, The Netherlands

^6^ Department of Aquatic Ecology and Environmental Biology, Institute for Water and Wetland Research (IWWR), Radboud University, Heyendaalseweg 135, 6525 AJ, Nijmegen, the Netherlands

*Corresponding author: Hacen El-Hacen ([e.h.m.el.hacen@rug.nl](mailto:e.h.m.el.hacen@rug.nl))

Postal address: P.O. Box 11103, 9700 CC Groningen, The Netherlands

Telephone/Fax: [+31] 50 363 5205

***Supplementary 1:* Witnessed *s*eagrass die-off events in Banc d’Arguin**

**
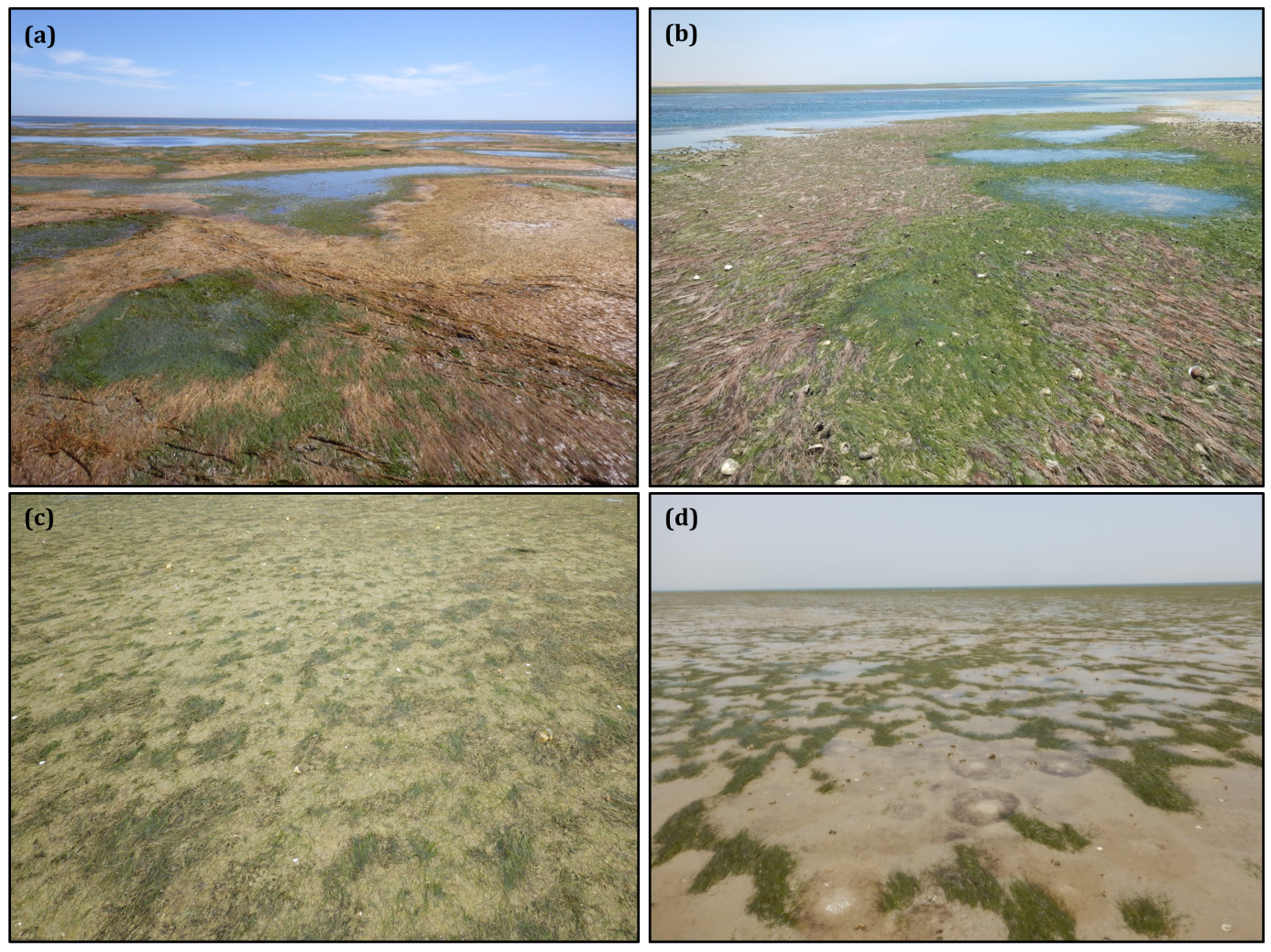
**

Figure S1. Landscape-scale seagrass die-off related events in Parc National du Banc d’Arguin, Mauritania (see Fig. 2) observed during the study period. Upper panels (a, b) show sudden die-off low on the elevational gradient due to unknown physiological damage to the tissues. Lower panels (c, d) demonstrate degrading seagrass higher on the elevational gradient du to massive deposition of mud-packages. Left photos (a, c) were taken in May 2015 and the right ones (b, d) on May 2016.

***Supplementary 2: Structural equation modelling procedures***

The process of conducting structural equation models typically involves model specification, identification, parameter estimates, evaluation of model fit, and model re-specification (Grace 2006, Grace *et al.* 2010). In the present study*, a prior* meta-model was developed, based upon our knowledge on the functioning of *Zostera* *noltii*, including important interactions that might affect its recovery (Fig. S2). This meta-model included eight observed exogenous variables and was improved by removing the non-significant paths from the meta-model^1,2^, and compared these models using Akaike’s information criterion (AICc)^3–7^. Our *prior* model depicted that elevation and die-off treatments would have direct effects on recovery rate as well as on abiotic and biotic variables that might affect the recovery rate of seagrass after die-off. Variables were checked for parametric assumptions and were transformed when necessary to meet the assumptions.


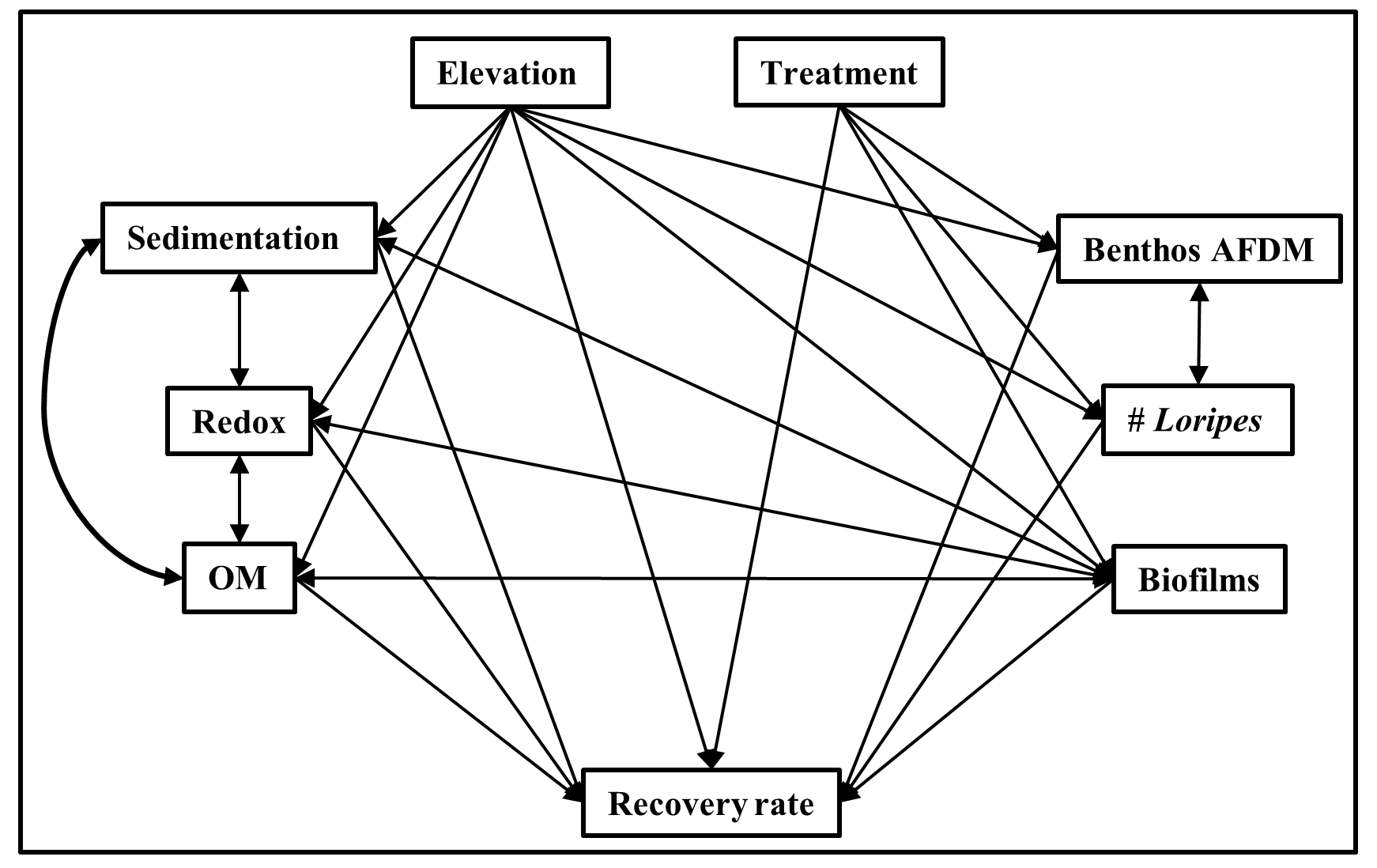


**Figure S2.** *A priori* meta-model of possible links of the predicted effects of elevation, treatment, and various abiotic and biotic factors on the recovery rate of seagrass after experimental die-off events at Zira, Banc d’Arguin.

Table S1. Complete outcomes of the fitted model tested in the Piecewise SEM showing standardized coefficients of the effects of elevation, die-off treatments, and various abiotic and biotic factors on the seagrass recovery after experimental die-off events at Banc d’Arguin, Mauritania. Significant paths (*P* < 0.05) are denoted in bold.

| **Response** | **Predictor** | **Estimate** | **SE** | **P-value** | **Std Estimate** |
| --- | --- | --- | --- | --- | --- |
| Redox | Elevation | 0.004 | 0.007 | 0.556 | 0.152 |
| Redox | Die-off size | 0 | 0.001 | 0.95 | -0.002 |
| Sedimentation | Elevation | 0.385 | 0.044 | **0.001** | **0.625** |
| Sedimentation | Die-off size | 0.158 | 0.059 | **0.008** | **0.154** |
| Organic matter | Elevation | 0.836 | 0.054 | **<0.001** | **0.903** |
| Organic matter | Die-off size | -0.029 | 0.048 | 0.554 | -0.019 |
| # Loripes | Elevation | -0.04 | 0.11 | 0.733 | -0.039 |
| # Loripes | Die-off size | 0.306 | 0.118 | **0.011** | **0.176** |
| Benthos AFDM | Elevation | 0.111 | 0.208 | 0.623 | 0.095 |
| Benthos AFDM | Die-off size | 0.067 | 0.135 | 0.619 | 0.035 |
| Benthos AFDM | Organic matter | 0.057 | 0.19 | 0.765 | 0.045 |
| Biofilm | Elevation | 0.032 | 0.335 | 0.929 | 0.023 |
| Biofilm | Die-off size | -0.015 | 0.055 | 0.787 | -0.006 |
| Biofilm | Sedimentation | -0.151 | 0.065 | **0.022** | **-0.106** |
| Biofilm | Organic matter | -0.189 | 0.076 | **0.014** | **-0.125** |
| Recovery rate | Redox | 0.082 | 2.283 | 0.972 | 0.002 |
| Recovery rate | Sedimentation | -0.075 | 0.049 | 0.128 | -0.034 |
| Recovery rate | Organic matter | 0.005 | 0.06 | 0.938 | 0.003 |
| Recovery rate | # Loripes | 0.009 | 0.027 | 0.732 | 0.007 |
| Recovery rate | Benthos AFDM | -0.039 | 0.024 | 0.116 | -0.034 |
| Recovery rate | Biofilm | -0.118 | 0.055 | **0.032** | **-0.128** |
| Recovery rate | Elevation | -0.409 | 0.294 | **<0.001** | **-0.306** |
| Recovery rate | Die-off size | -0.218 | 0.041 | 0.236 | -0.098 |
| Sedimentation | Organic matter | -0.114 | NA | 0.935 | -0.114 |
| Sedimentation | Redox | -0.018 | NA | 0.597 | -0.018 |
| Redox | Organic matter | 0.086 | NA | 0.125 | 0.086 |
| # Loripes | Benthos AFDM | 0.313 | NA | **<0.001** | **0.313** |
| # Loripes | Redox | 0.096 | NA | 0.1 | 0.096 |
| *Notes*: Estimate = standardized path coefficient; SE = standard error; | | | | | |
| Std Estimate = conditional R^2^; Model Fisher's C statistic = 14.65; *P* = 0.56. | | | | | |

***Supplementary 3: Macrofaunal recolonization***


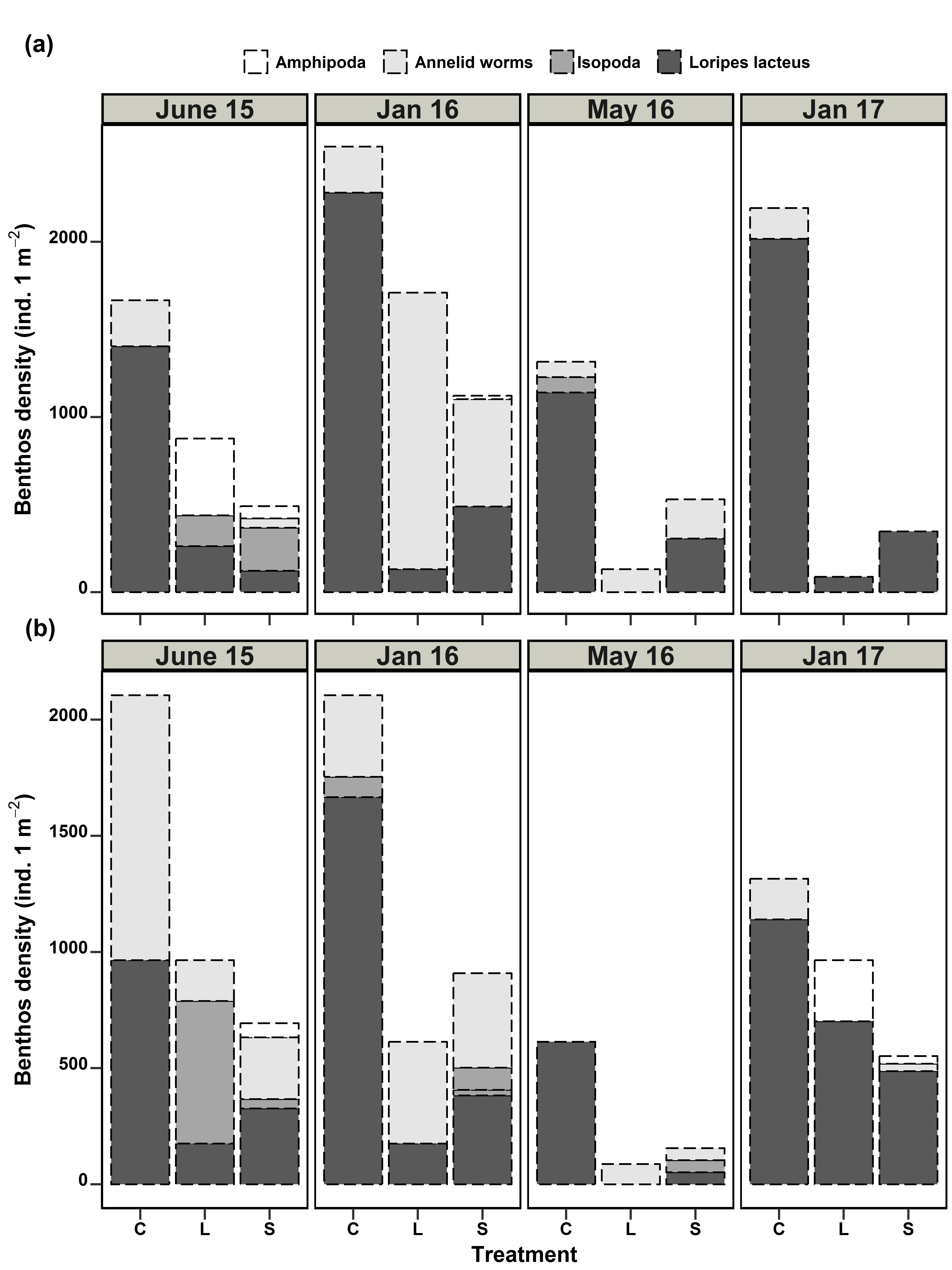


**Figure S3.** Seasonal change in benthos density (m^-2^) of the most common species (94%) found in the experimental plots (C = control, L = large, S = small) along an elevation gradient, (a) high zone and (b) low zone.


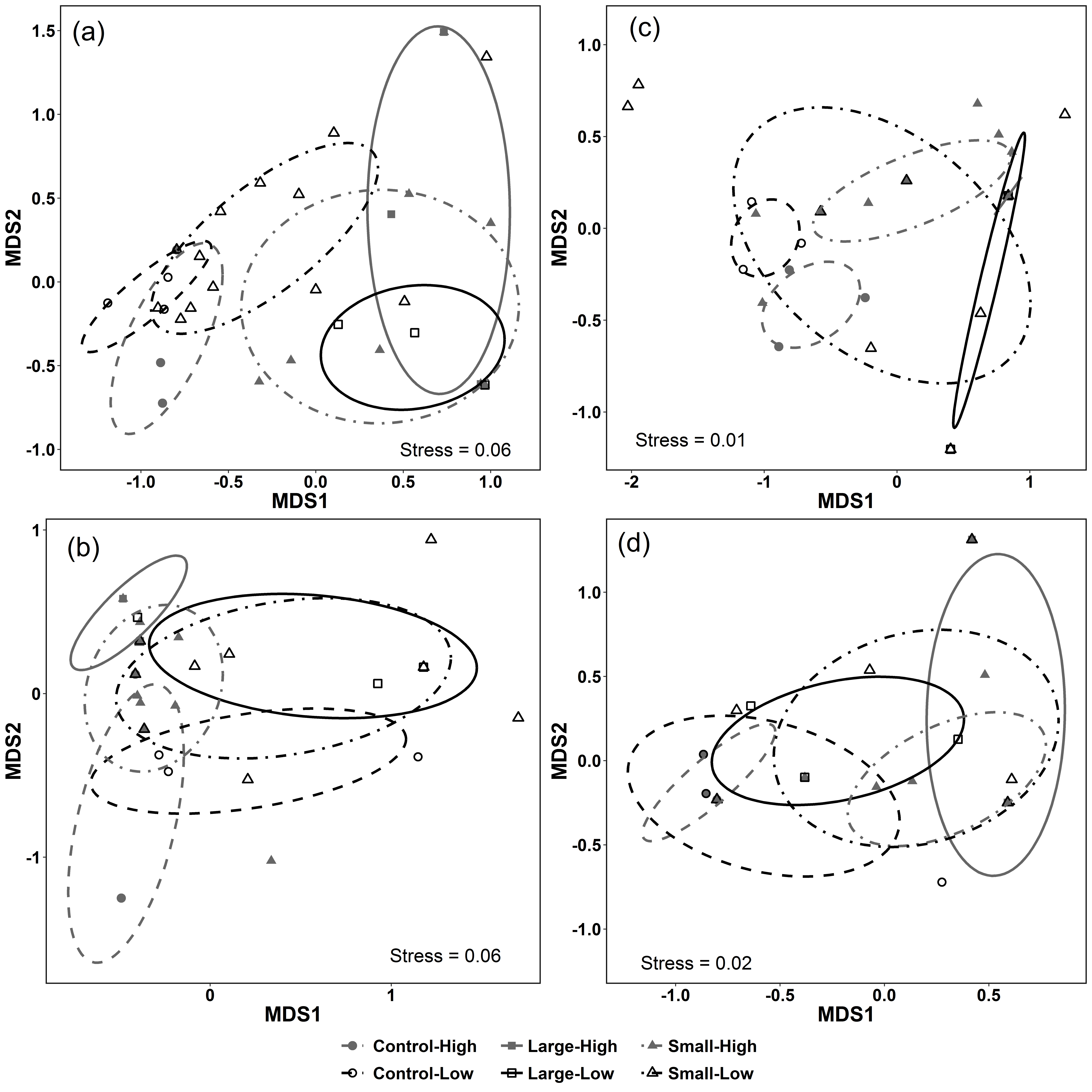


**Figure S4.** MDS plots showing differences in macrobenthic community assemblages between disturbed plots (large, small) and controls in two elevational zones (high, low) represented by 95% confidence interval ellipses. (a) six months, (b) 12 months, (c) 18 months, and (d) 24 months after defaunation.

**References**

1. Grace, J. B. *Structural equation modeling and natural systems*. (Cambridge University Press, Cambridge, 2006).

2. Grace, J. B., Anderson, T. M., Olff, H. & Scheiner, S. M. On the specification of structural equation models for ecological systems. *Ecological Monographs* **80,** 67–87 (2010).

3. Edgar, G. J. *et al.* Abundance and local-scale processes contribute to multi-phyla gradients in global marine diversity. *Science Advances* **3,** e1700419 (2017).

4. Lefcheck, J. S. piecewiseSEM : Piecewise structural equation modelling in r for ecology, evolution, and systematics. *Methods in Ecology and Evolution* **7,** 573–579 (2016).

5. Lefcheck, J. S. & Duffy, J. E. Multitrophic functional diversity predicts ecosystem functioning in experimental assemblages of estuarine consumers. *Ecology* (2015).

6. Lefcheck, J. S. *et al.* Long-term nutrient reductions lead to the unprecedented recovery of a temperate coastal region. *Proceedings of the National Academy of Sciences* (2018).

7. Rosseel, Y. lavaan : An R Package for Structural Equation Modeling. *Journal of Statistical Software* **48,** 1–36 (2012).
